# Light influences the effect of exogenous ethylene on the phenolic composition of Cabernet Sauvignon grapes

**DOI:** 10.3389/fpls.2024.1356257

**Published:** 2024-02-23

**Authors:** Meiying Liu, Qinggang Zhu, Yanhong Yang, Qianqian Jiang, Hui Cao, Zhenwen Zhang

**Affiliations:** ^1^ Key Laboratory of Biochemistry and Molecular Biology in University of Shandong, School of Advanced Agricultural Sciences, Weifang University, Weifang, China; ^2^ College of Enology, Northwest A&F University, Yangling, Shaanxi, China; ^3^ College of Horticulture, Northwest A&F University, Yangling, Shaanxi, China

**Keywords:** light, exogenous ethylene, anthocyanins, non-anthocyanins, grape berries

## Abstract

The gaseous phytohormone ethylene (ETH) plays a key role in plant growth and development, and is a major regulator of phenolic biosynthesis. Light has long been known to influence phytohormone signaling transduction. However, whether light influences the effect of ETH on the phenolic composition of grapes (*Vitis vinifera* L.) is an open question. Here, the accumulation and composition of anthocyanins and non-anthocyanin phenolics were analyzed in Cabernet Sauvignon grapes under four treatments: light exposure with and without ETH treatment, and box-shading with and without ETH treatment. Both light and ETH promoted ripening, decreased the color index (L*, C*, and h*), and accelerated the color change from green to red and purplish red. Sunlight-exposed grapes had the highest contents of most anthocyanins, flavonols, flavan-3-ols, and hydroxybenzoic acids. In addition, light exposure increased the ratios of 3’5’-substituted/3’-substituted anthocyanins and flavonols, but decreased the ratios of methoxylated/non-methoxylated and acylated/non-acylated anthocyanins and flavan-3-ols. Notably, the effects of ETH were influenced by light exposure. Specifically, ETH treatment promoted anthocyanin and non-anthocyanin biosynthesis in light-exposed grapes, and their increasing multiples were remarkably higher under light-exposed conditions. Furthermore, ETH treatment decreased the ratios of methoxylated/non-methoxylated, 3’5’-substituted/3’-substituted, and acylated/non-acylated anthocyanins and flavan-3-ols in light-exposed grapes, each of which was increased by ETH treatment in shaded grapes. Fifteen differential phenolic components were identified through partial least-squares-discriminant analysis (PLS-DA). Among them, cyanidin-3-*O*-(cis-6-*O*-coumaryl)-glucoside, petunidin-3-*O*-(6-*O*-acetyl)-glucoside, petunidin-3-*O*-(trans-6-*O*-coumaryl)-glucoside, petunidin-3-*O*-glucoside, myricetin-3-*O*-galactoside, kaempferol-3-*O*-galactoside, and kaempferol-3-*O*-glucoside were the main differential components between ETH treatments under different light conditions. This study contributes to the understanding of the impact of ethylene treatment under dark and light conditions on phenolic synthesis in grape berries.

## 1 Introduction

Phenolic compounds, including anthocyanins, flavonols, flavanols, and phenolic acids, are the most abundant secondary metabolites in grapes (*Vitis vinifera* L.) and are important determiners of wine quality, affecting both color and sensory properties (e.g., astringency and bitterness). In grapes, each of these compounds performs important physiological functions such as serving as pigments or co-pigments, scavenging free radicals, and protecting against ultraviolet (UV) radiation as well as bacterial and fungal pathogens (Williams et al., 2000), also their contributions to free radical defense in human health have been reported over years (Ferrer-Gallego et al., 2014; Ky et al., 2014; Valdés et al., 2015).

The phytohormone ethylene (ETH) plays a key role in vegetative development, abscission, senescence, reproduction, stress response, and fruit ripening (Liu et al., 2015a). For fruits, the ripening process is normally viewed distinctly in climacteric and non-climacteric fruits because of the dramatic increase in the rate of respiration and ethylene production during ripening in climacteric fruits, thus ethylene plays a major role in the ripening process of climacteric fruits (Paul et al., 2012). Grapes are classified as non-climacteric fruits and appear to ripen independently of ETH production. However, some studies report that a transient increase in endogenous ETH production may control berry development, accompanying critical ripening processes such as anthocyanin accumulation, sugar production, and decreasing acidity (Chervin et al., 2004; Tira-Umphon et al., 2007). In addition, the expression of structural genes related to phenylpropanoid and flavonoid biosynthesis, as well as their transcriptional regulators, is enhanced by exogenous ETH treatment (Liu et al., 2016).

Light also influences the development, ripening, and phenolic composition of grape berries, and ultimately affects wine quality (Downey et al., 2006). For example, grapes exposed to sunlight exhibit increased contents of anthocyanins and flavonols compared with shaded fruits (Spayd et al., 2002; Downey et al., 2008). Furthermore, these light-driven increases in phenolic compound production are correlated with increased expression of structural genes related to flavonoid biosynthesis, as well as their transcriptional regulators (Koyama and Goto-Yamamoto, 2008; Sun et al., 2017).

Recent research suggests that light signaling affects phytohormone biosynthesis and signal transduction, including for ETH, auxin (AUX), gibberellic acid (GA), cytokinins (CTKs), and brassinosteroids (BRs) (Carvalho et al., 2011).To date, a considerable amount of research effort has been focused on the interaction between light and ETH signaling, most notably the effect of light on the triple response. In *Arabidopsis* seedlings, ETH-induced stimulation of hypocotyl elongation is mediated by light. Application of 1-aminocyclopropane-1-carboxylic acid (ACC; a precursor of ETH) suppresses hypocotyl elongation under dark conditions, but stimulates hypocotyl elongation upon exposure to light (Smalle et al., 1997; Zhong et al., 2009; Liang et al., 2012). In addition, light may act as a negative regulator in the destabilization of EIN3/EIL1, a transcription factor (TF) involved in ETH signal transduction (Lee, 2006; Liang et al., 2012). Similarly, various components of ethylene signaling, including a member of serine/threonine-protein kinase CTR1, a negative regulator of the ethylene response pathway and ACO, the last enzyme in the ethylene production pathway which controlled the biosynthesis of ethylene in plants, were significantly affected in the light-exposed berries during development (Sun et al., 2017). This suggests that there is a reciprocal interaction between these two signaling pathways.

Since, ETH treatment results in accelerated ripening and flavonoid accumulation, and these phenomena are correlated with enhanced ETH signal transduction in grapes (Chervin and Deluc, 2010; Liu et al., 2016), the interactions between light and ETH signaling may regulate the biosynthesis of phenolic compounds during berry development, and thus studies should be conducted on the relationship between phenolic composition, light, and ETH. Here, we studied the accumulation and composition of phenolic acids, flavanols, anthocyanins, and flavonols in Cabernet Sauvignon grapes exposed to different combinations of light and exogenous ETH. These analyses would provide additional data for a comprehensive understanding of the events underlying the color changes in ethephon-treated berries in dark and light and would broaden our understanding of the response of berries to environmental influences.

## 2 Materials and methods

### 2.1 Experimental field site

The field experiment was conducted in a commercial vineyard in Jingyang, Shaanxi, China (34°65′N, 108°75′E). This region contains hilly and semi-hilly terrain and has a semi-humid, continental monsoon climate with 2195.2 h of sunshine annually and an average frost-free period of 213 d. The mean annual temperature and precipitation are 13°C and 548.7 mm, respectively. The vineyard soil is classified as sandy loam. All agricultural operations followed standard commercial practices and were identical for all experimental vines.

### 2.2 Experimental design and berry sampling

Samples were selected from grapevines (*Vitis vinifera* L cv. Cabernet Sauvignon) colonized in 2009 and grafted onto their own root-stocks. All vines were in a Vertical Shoot Position training system orientated east-west with short shoot pruning and each shoot had 1~2 clusters. Row orientation has a pronounced effect on the amount of photo-synthetically active radiation (PAR) received by the two sides of the rows. Grape clusters were thus selected from the southern sides of rows to maintain a consistent PAR.

At the onset of véraison (colored of 5~10% of the berries, definitely on the 60 days after flowering (DAF)), the clusters were dipped into a 400 mg/L solution of ethephon (>85% purity; Sangon, Inc., Shanghai, China) containing a buffered wetting agent (1 ml/L Tween 80), defined into +E (no rain for two weeks after treatment). Control clusters were dipped into the wetting agent only, defined into -E. After that, the treated berries separated into two groups, normal light-exposure treated berries, clustered into +L, and the other group used polypropylene boxes modelled on the design by Downey et al. (2008) to cover the clusters immediately after ethephon dipping, defined into -L, and the shaded clusters remained enclosed until harvest (**Supplementary Figure 1**). Thus, this research designed three replicates, and each received four treatments: light exposure with (+L+E) and without (+L-E) ethephon, and box-shading with (-L+E) and without (-L-E) ethephon. And for each treatment in each replicate, twenty grape clusters in the same side, same position and in the same size were selected from ten plants.

**Figure 1:**
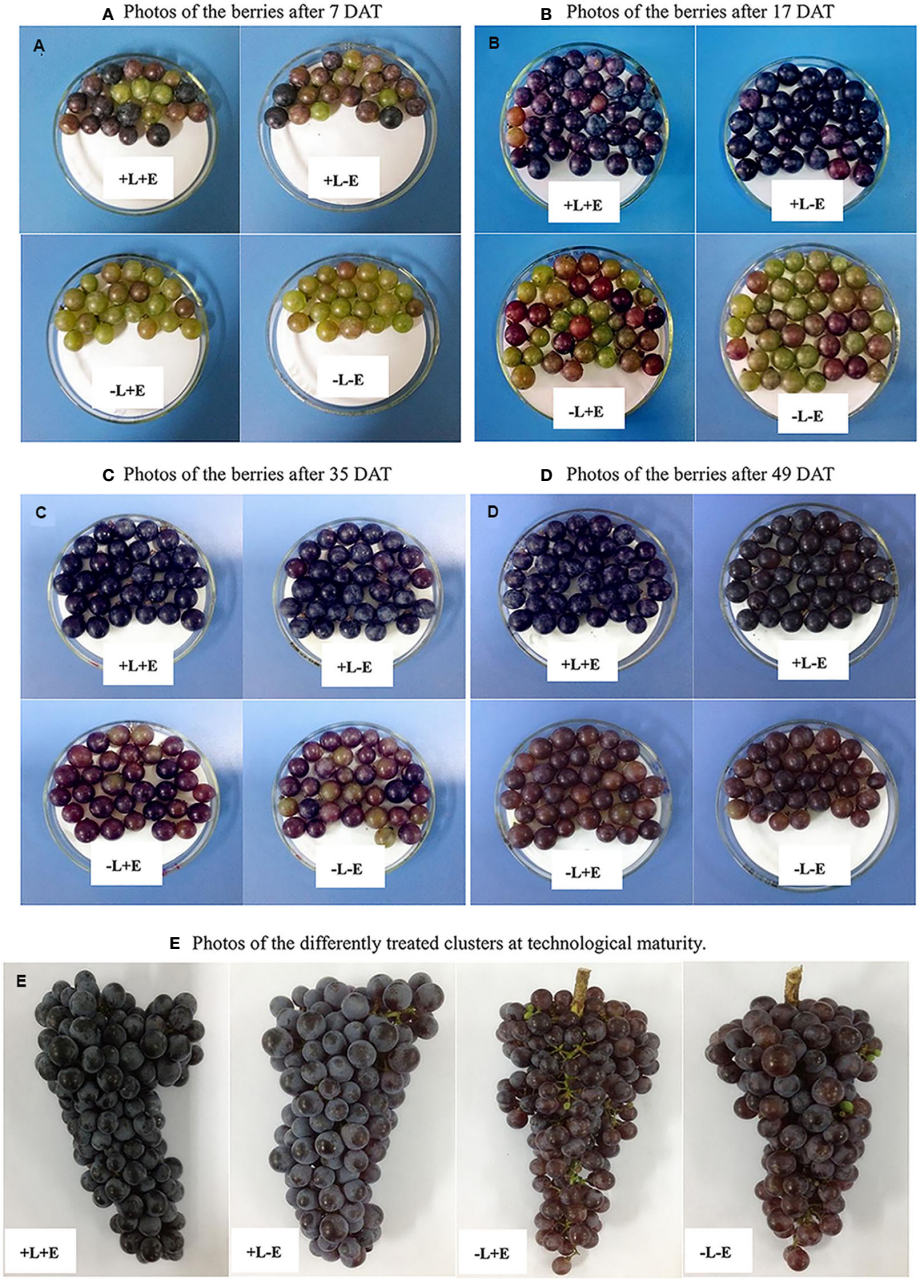
The Grape samples collected at 7, 17, 35, and 49 DAT.

Grape samples of seventy berries for each replicate of each treatment were randomly collected at 7, 17, 21, 28, 35, 49 DAT (days after treatments), and used for measurement of the ripening parameters and the CIE L* a* b* color index. Besides, a total of 100 berries of each replicate were randomly sampled at 7, 17, 35, 49 days after treatment, corresponding to the half-colored, fully-colored, half-ripe, and fully ripe of the berries for the analysis of the phenolic compounds (as shown in **Figure 1**). After sampling, the skins of 100 berries were manually peeled, vacuum freeze-dried and weighted, then ground in liquid nitrogen, and stored at -80°C for the extraction of phenolic compounds.

### 2.3 Measurement of the ripening parameters

The ripening parameters were defined by soluble solid content and pH, and fifty berries were randomly sampled from each experimental cluster at each sampling date, berry juice was obtained by manual pressing. Total soluble solid content (Brix) was determined using a PAL-1 digital refractometer (Atago, Tokyo, Japan). The berry pH was measured using a pH meter (model PB-10, Sartorius, Germany).

### 2.4 Determination of the CIE L* a* b* color index

Twenty randomly sampled berries were measured to determine the CIE L* a* b* color index as previously described (Rolle and Guidoni, 2007) using a Minolta CR400 reflectance colorimeter (Konica Minolta, Tokyo, Japan), and the equations C* (chroma) = (a*^2^+ b*^2^) ^1/2^ and h* (hue) = tan^-1^ (b*/a*) were used to calculate to the C* and h* (Nour et al., 2015), and the lightness (L*) represent whether the color is closer to black (low values) or white (high values), hue (h*) is the perceived color of the fruit: (range 0°-360°, in order as follows: red, yellow, green, blue), and chroma (C*) describes the saturation of a color (CIE Publication, 2004). Before used, standard chromaticity (L*= 97.06, a*=0.04, b* =2.01) was used to calibrate reflectance colorimeter.

### 2.5 HPLC-DAD/ESI-MS analysis of anthocyanin compounds

For anthocyanins, triplicated 0.5g freeze-dried skin power into 50 mL centrifuge tube with 10 mL solvent (methanol/water/acetic acid, 70:29:1, v/v/v) in an orbital shaker at 300 rpm for 100 min at 25°C. After pouring out the supernatant, the precipitate was re-extracted with the same solvent (10 mL) three times. The supernatants were combined in a 50 mL tube, and centrifuged at 8,000 rpm for 5 min. Finally, the supernatants were collected and filtered through a 0.45-μm organic membrane. Finally, the filtrates were used for qualitative and quantitative analyses of HPLC-DAD/ESI-MS.

The anthocyanins were chromatographically analysed using an Agilent 1100 series LC-MSD trap VL (Agilent, Santa Clara, CA, USA) equipped with a G1379A degasser, G1312BA Quatpump, G1313A ALS autosampler, G1316A column, G1315A DAD, and reversed-phase column (Kromasil C18, 250 × 4.6 mm id, 5 μm particle size, Restek corporation, Bellefonte, PA, USA). The mobile phase A was 6% (v/v) acetonitrile and 2% (v/v) formic acid in water, and B was 54% (v/v) acetonitrile containing 2% (v/v) formic acid in water. The proportions of solvent B varied as follows: 1–18 min, 10%–25%; 18–20 min, 25%; 20–30 min, 25%–40%; 30–35 min, 40%–70%; and 35–40 min, 70%–100%. The column was held at 50°C and was flushed at a flow rate of 1.0 mL·min^−1^. The injection volume was 30 μL, and the detection wavelength was 525 nm. MS conditions were: electrospray ionisation (ESI) interface, positive ion model, 30 psi nebuliser pressure, 12 mL·min^−1^ dry gas flow rate, 300°C dry gas temperature, and scans between m/z 100 and 1500 (Cheng et al., 2014; Liu et al., 2015b).

All anthocyanin compounds were identified by comparing their order of elution and retention time with those of standards, and the weight of molecular ion and the fragment ion were compared with standards and references (Villiers et al., 2004). Quantitative determinations used the external-standard method with commercial standards. The calibration curves were obtained by injection of standard solutions over a certain range of concentrations under the same conditions as the samples analysed (**Supplementary Table 1**). The compounds for which of no standards available were quantified with the curves of homologous non-acylated anthocyanins. the content of each anthocyanin compound was thus respectively expressed as relative dephinidin-3-*O*-glucoside, cyanidin-3-*O*-glucoside, petunidin-3-*O*-glucoside peonidin-3-*O*-glucoside, and malvidin-3-*O*-glucoside equivalence microgramme in per berry. All analyses were performed in triplicate.

### 2.6 HPLC-DAD/ESI-MS analysis of non-anthocyanin compounds

For non-anthocyanin phenolics (including flavanols, flavonols, phenolic acids), triplicate samples of 2.0g freeze-dried skin power were exhaustively extracted four times with 5mL of distilled water and 45mL of ethyl acetate in an orbital shaker (SHZ-88A, Taicang Experiment Equipment Factory, Jiangsu, China) for 30 min at 20°C. Then, these organic phases were combined and evaporated to dryness in a rotary evaporator (SENCO-R series; Shanghai Shensheng Biotech Co. Ltd., Shanghai, China) at 35°C under vacuum. Subsequently, the dried residuals were re-dissolved in 2 mL of methanol (HPLC grade). This methanol solution was filtered through a 0.45-μm organic membrane and analyzed by high performance liquid chromatography (HPLC) coupled with diode array detector (DAD) and electrospray ionization mass spectrometry (ESI-MS).

The chromatographic analyses of non-anthocyanins were performed using an Agilent 1200 series LC-MSD trap XCT (Agilent Corporation, Santa Clara, CA, USA) equipped with a G1322A Degasser, a G1312B Bin pump, a G1367C HiP-ALS autosampler, a G1316B TCC (thermostated column compartment), a G1314C VWD (variable wavelength detector) and a reversed phase column (ZORBAX Molecules 2013, 18 393 SB-C18, 3 × 50 mm i.d., 1.8 μm). The mobile phase consisted of (A) 1% acetic acid in water solution, and (B) 1% acetic acid in acetonitrile solution. The elution profile had the following proportions (v/v) of solvent B: 0.00–5.00 min, 5–8%; 5.00–7.00 min, 8–12%; 7.00–12.00 min, 12–18%; 12.00–17.00 min, 18–22%; 17.00–19.00 min, 22–35%; 19.00–21.00 min, 35–100%; 21.00–25.00 min, 100%; 25.00–27.00 min, 100–5%;. The column was held at 25°C and was flushed at a flow rate of 1.0 mL min^−1^. The injection volume was 2 µL and analyses were detected at 280 nm. MS conditions were as follows: Electrospray ionization (ESI) interface, negative ion model, 35 psi nebulizer pressure, 10 mL min^−1^ dry gas flow rate, 325°C dry gas temperature, and scans and scans at 100–1,500 m/z (Luan et al., 2013).

The calibration curves for non-anthocyanin standards were obtained in the same way as for anthocyanins by injecting standard solutions, and each identified substance was quantified using the curves of homologous non-anthocyanins (**Supplementary Table 1**) and expressed as microgramme in per berry.

### 2.7 Chemical and standards

All standards (showed in **Supplementary Table 1**) were purchased from Sigma-Aldrich Co. (St. Louis, MO, USA). HPLC-grade methanol, formic acid, and acetonitrile were purchased from Fisher (Fairlawn, NJ, USA). Analytical-grade methanol, formic acid, acetone and sodium acetate were purchased from the Beijing Chemical Reagent Plant (Beijing, China).

### 2.8 Statistical analysis

The data were analyzed using Microsoft Excel 2010 and were represented as mean of the triplicate experiments. One-way analyses of variance (ANOVAs) and Duncan’s multiple range tests were analyzed by DPS 7.55 to determine the significance of the difference among samples, with a significance difference at 0.05 level. Comparative heatmap, principal component analysis (PCA) and partial least square discriminant analysis (PLS-DA) were performed by Metabo-Analyst. (http://www.metaboanalyst.ca/MetaboAnalyst/faces/home.xhtml), and auto-scaling was used in normalization procedure.

## 3 Results

### 3.1 Effects of light and ethylene on grape ripening

**Figure 2:**
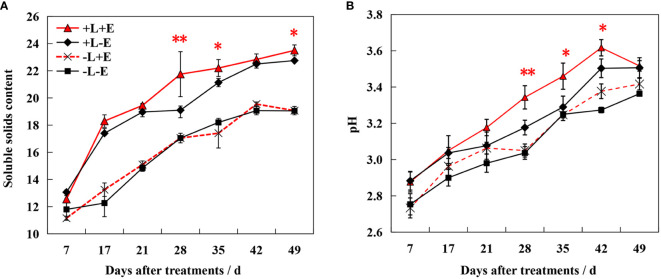
Changes of Brix value **(A)** and pH **(B)** in grape berry of different treatments. The red asterisk indicates a significant difference between the ETH-treated light-exposed (+L+E) samples and the untreated light-exposed (+L-E) samples. Similarly, the black asterisk indicates a significant difference between the ETH-treated shaded (-L+E) samples and the untreated shaded (-L-E) samples. One asterisk and two asterisks represent significance at the 0.05 and 0.01 levels, respectively.

The sugar content increased gradually during ripening, with shading (-L+E and -L-E) significantly inhibiting sugar accumulation (**Figure 2**). At 49 days after treatment (DAT), light-exposed ETH-treated (+L+E) grapes exhibited a 2.83% higher sugar content than light-exposed untreated (+L-E) grapes. However, shaded ETH-treated (-L+E) grapes exhibited only a 1.24% increase in sugar content compared to shaded untreated (-L-E) grapes. Overall, the increase range of the sugar content by ETH treatment in light-exposed grapes (+L+E vs +L-E) was significantly higher compared to ETH treatment in shaded grapes (-L+E vs -L-E). Additionally, both ETH (+E) and light (+L) significantly increased berry pH, with ETH treatment affecting light-exposed grapes more dramatically than shaded grapes.

### 3.2 Effects of light and ethylene on grape skin color

**Figure 3:**
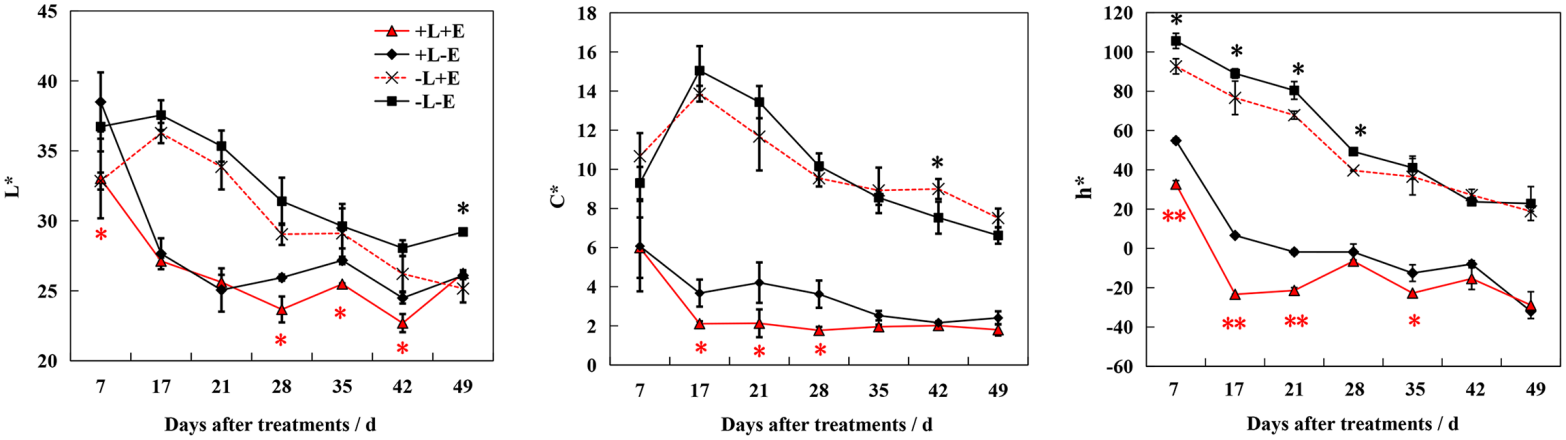
Changes of color indicators L*、C*、h* in grape berry of different treatments. The red asterisk indicates a significant difference between the ETH-treated light-exposed (+L+E) samples and the untreated light-exposed (+L-E) samples. Similarly, the black asterisk indicates a significant difference between the ETH-treated shaded (-L+E) samples and the untreated shaded (-L-E) samples. One asterisk and two asterisks represent significance at the 0.05 and 0.01 levels, respectively.

During ripening, the color of the berry skin gradually deepened, first turning red and then nearly black. To comprehensively evaluate this color change process, we measured the three coordinates of the CIE color system (L*, a*, and b*) and converted them to L*, C*, and h* (**Figure 3**). After treatment, lightness (L*), chroma (C*), and hue angle (h*) decreased over the course of berry development. Both ETH (+E) and light (+L) consistently promoted decreasing L*, C*, and h*, with light exposure (+L) resulting in a more dramatic effect. Compared to ETH-untreated grapes (+L-E and -L-E), these parameters decreased more significantly in light-exposed ETH-treated (+L+E) grapes than in shaded ETH-treated (-L+E) grapes. These results indicate that ETH was more effective at accelerating the ripening-associated color change in light-exposed grapes.

### 3.3 Effects of light and ethylene on grape skin anthocyanin composition

**Figure 4:**
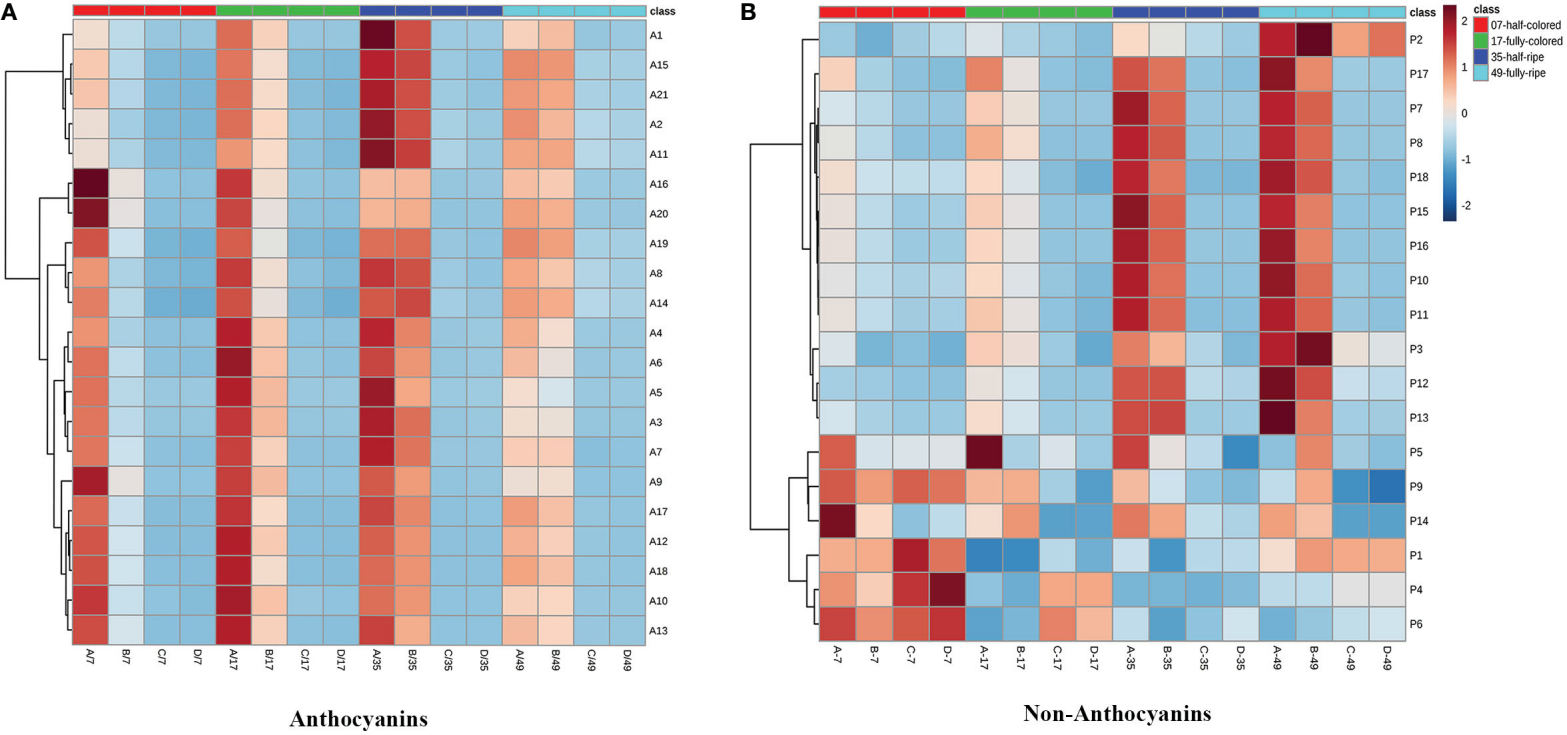
The heatmap of the contents of individual anthocyanins **(A)** and non-anthocyanins **(B)** between different treatments. A, B, C, and D on the horizontal axes represent four treatments: +L+E, +L-E, -L+E, and -L-E, respectively. The A1-A20 on the horizontal axis of **Figure 2A** represent each individual anthocyanin compound, respectively, and the contents and full names are provided in **Supplementary Table 2**, A21 indicates the total content of anthocyanin compounds. The P1-P18 on the horizontal axis of **Figure 2B** represent each non-anthocyanin compound, respectively, and the contents and full names are provided in **Supplementary Table 3**, P19 indicates the total content of non-anthocyanin compounds.

Twenty anthocyanin compounds were detected in grape skins throughout berry development, including malvidin-3-*O*-glucoside, peodidin-3-*O*-glucoside, petunidin-3-*O*-glucoside, cyanidin-3-*O*-glucoside, and dephinidin-3-*O*-glucoside, as well as their acetylated and coumarylated derivatives (**Supplementary Tables 2**, **4**). The most abundant compounds were malvidin-3-*O*-(6-*O*-acetyl)-glucoside (A11) and malvidin-3-*O*-glucoside (A15), followed by malvidin-3-*O*-(*trans*-6-*O*-coumaryl)-glucoside (A2) and petunidin-3-*O*-glucoside (A17). MetaboAnalyst was used to develop a heat map of the contents of anthocyanin compounds (**Figure 4A**). Within the map, contents of the same individual anthocyanin under different treatments were normalized, with A, B, C, and D representing +L+E, +L-E, -L+E, and -L-E, respectively. Compared to shaded grapes (-L; C and D), light-exposed grapes (+L; A and B) contained significantly higher contents of all anthocyanin compounds (**Figure 4A**). Differences between ETH-treated and untreated grapes were also more apparent, as shown, compared with untreated light-exposed grapes (+L-E; B), ETH-treated light-exposed grapes (+L+E; A) contained significantly higher contents of most anthocyanins, especially cyanidin-3-*O*-(6-*O*-acetyl)-glucoside (A16), cyanidin-3-*O*-glucoside (A20), cyanidin-3-*O*-(*cis*-6-*O*-coumaryl)-glucoside (A9), cyanidin-3-*O*-(*trans*-6-*O*-coumaryl)-glucoside (A10), and dephinidin-3-*O*-(6-*O*-acetyl)-glucoside (A13) and these promotional effects were most obvious shortly after treatment. However, there were few significant differences observed between untreated (-L-E; D) shaded grapes and ETH-treated (-L+E; C) shaded grapes.

### 3.4 Effects of light and ethylene on grape skin non-anthocyanin composition

In addition, seventeen individual non-anthocyanin phenolics were detected in grape skins during berry development, including five flavanols, eleven flavonols, and one *p*-hydroxybenzoic acid (**Supplementary Table 3**). The most abundant non-anthocyanin compounds were quercetin-3-*O*-glucuroside (P9) and quercetin-3-*O*-glucoside (P11), followed by procyanin B1 (P1) and myricetin-3-*O*-glucoside (P8). MetaboAnalyst was used to develop a heat map of the contents of non-anthocyanin phenolic compounds (**Figure 4B**). Compared to shaded grapes (-L; C and D), light-exposed grapes (+L; A and B) contained significantly higher contents of most flavonol compounds and trihydroxylated flavanols like gallocatechin (P2) and epigallocatechin (P3). However, the contents of procyanin B1 (P1), procyanin C1 (P6), and catechin (P4) were slightly decreased by light exposure. Besides, compared with untreated light-exposed grapes (+L-E; B), ETH-treated light-exposed grapes (+L+E; A) contained significantly higher contents of most non-anthocyanins, including total non-anthocyanins (P18), myricetin (P17), quercetin-3-*O*-rhamnoside (P14), and kaempferol-3-*O*-galactoside (P15), and also there were few significant differences observed between untreated (-L-E; D) and ETH-treated (-L+E; C) shaded grapes.

### 3.5 Analysis and characterization of different samples based on the phenolic variables

**Figure 5:**
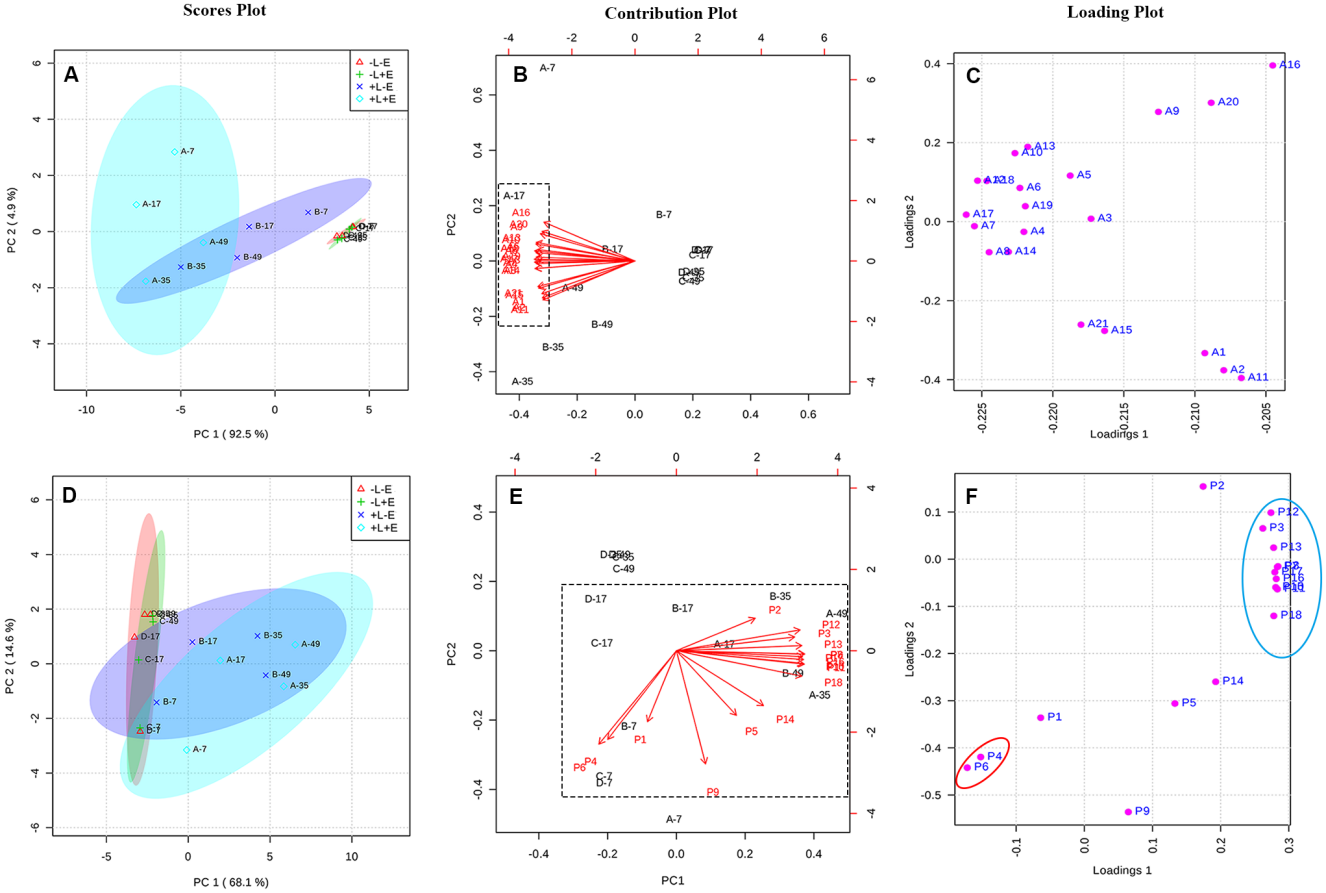
The PCA analysis of the contents of anthocyanin and non-anthocyanin compounds between different treatments. Panel **(A–C)** are respectively, the scores plot, contribution plot and loading plot for the anthocyanin compounds, and Panel **(D–F)** are respectively, the scores plot, contribution plot and loading plot for the non-anthocyanin compounds.

Principal component analysis (PCA) was used to explore and easily visualize the differences between samples, and to further examine the effects of light and ETH on the compositions of anthocyanins in grape skins (**Figures 5A–C**). Principal component 1 (PC1) explained 92.5% of the variance and separated ETH-treated light-exposed (+L+E; A) from untreated light-exposed (+L-E; B) grapes (**Figure 5A**). All variables associated with ETH treatment under light exposure (+L+E) were located in the negative half of the X-axis, primarily due to the higher contents of malvidin-3-*O*-glucoside (A15), peodidin-3-*O*-glucoside (A17), petunidin-3-*O*-glucoside (A19), cyanidin-3-*O*-glucoside (A20), dephinidin-3-*O*-glucoside (A18), and their acetylated and coumarylated derivatives (A13, A10, A6, A5, A3, A7, A14, A16, A8, and A18) in ETH-treated light-exposed (+L+E) grapes compared to untreated light-exposed (+L-E) grapes and ETH-treated shaded (-L+E) grapes (**Figures 5B, C**). These results suggest that anthocyanin biosynthesis is upregulated by both light exposure and ETH treatment under light exposure. However, no separation was observed between ETH-treated shaded (-L+E) grapes and untreated shaded (-L-E) grapes, possibly because anthocyanin biosynthesis was not significantly influenced by ETH treatment under dark conditions.

PCA was also performed to examine the effects of light and ETH on the compositions of non-anthocyanin phenolics in grape skins (**Figures 5D–F**). In this case, PC1 explained 68.1% of the variance, and separated the light-exposed (+L+E and +L-E) grapes from shaded (-L+E and -L-E) grapes (**Figure 5D**). All variables associated with shading (-L+E and -L-E) were located in the negative half of the X-axis, primarily due to the higher contents of catechin (P4) and procyanin P1 (P6) in light-exposed (+L+E and +L-E) grapes than in shaded (-L+E and -L-E) grapes (**Figures 5E, F**). In addition, individual non-anthocyanin phenolics were located at the maximum position of the positive half of the X and Y axes, including syringetin-3-*O*-glucoside (P12), epigallocatechin (P3), isorhamnetin-3-*O*-glucoside (P13), and quercetin 3-*O*-glucuroside (P9), among others. These compounds were significantly more abundant in light-exposed (+L) grapes than in shaded (-L) grapes (as showed in **Supplementary Table 3**). Notably, ETH-treated light-exposed (+L+E; A) grapes and untreated light-exposed (+L-E; B) grapes exhibited a higher degree of separation than the ETH-treated shaded (-L+E) grapes and untreated shaded (-L-E) grapes (**Figure 5D**).

According to the PCAs, both light and ETH significantly affected the contents of anthocyanins and non-anthocyanins in grape skins. In addition, exposure to light modulated the ETH treatment, particularly its effect on anthocyanin composition.

### 3.6 Effects of light and ethylene on the ratios of different phenolic compounds in grape skins

**Figure 6:**
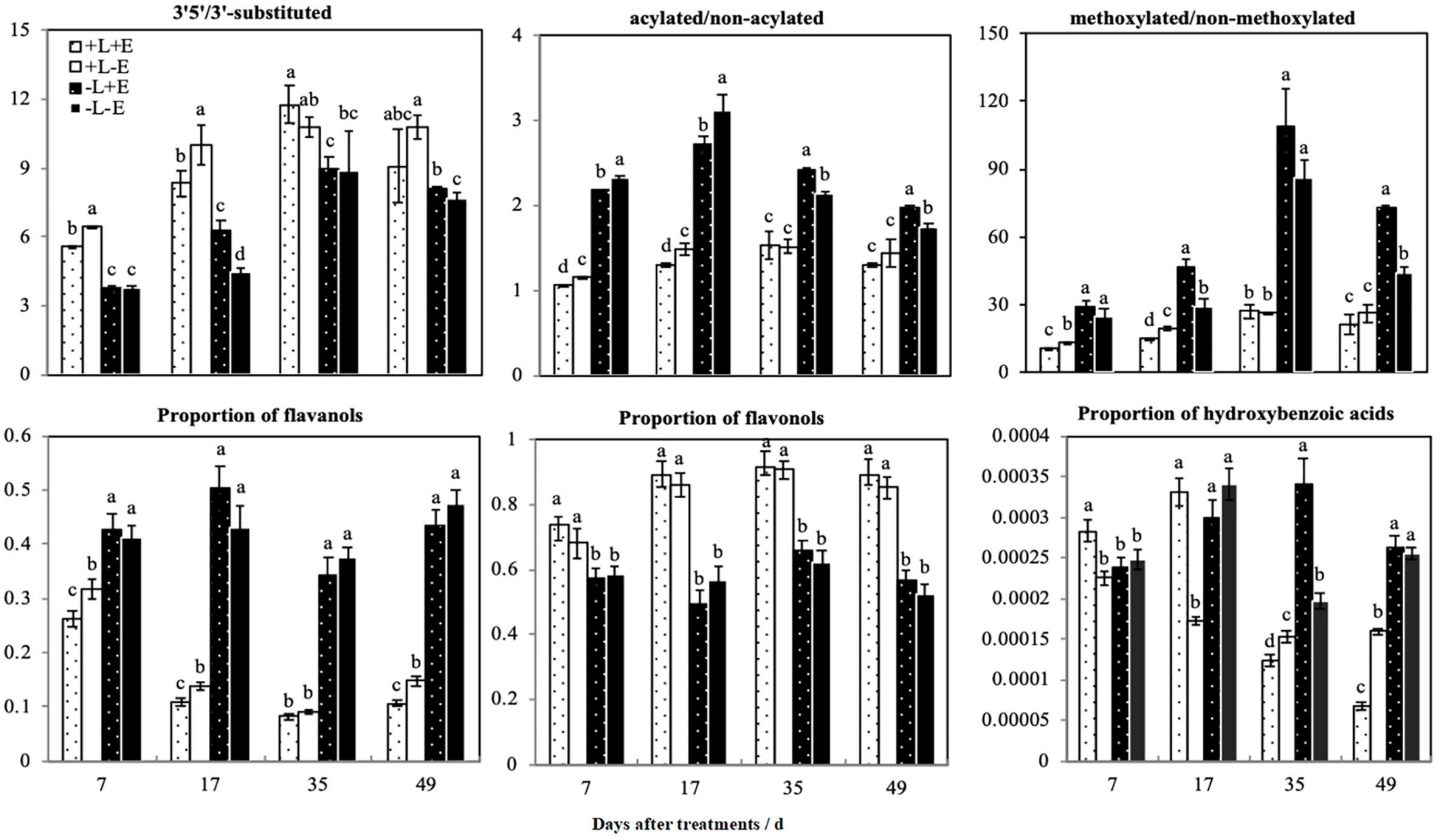
The ratios of different patterns of phenolic compounds between different treatments. a and b indicate significant differences at *P* < 0.05.

We calculated the ratios of different modified anthocyanins (e.g., 3’5’-substituted/3’-substituted, methoxylated/non-methoxylated, and acylated/non-acylated) in grape skins (**Figure 6**). Compared with shaded (-L) grapes, light-exposed (+L) grapes exhibited an increased ratio of 3’5’-substituted/3’-substituted anthocyanins but decreased ratios of methoxylated/non-methoxylated and acylated/non-acylated anthocyanins. However, in light-exposed grapes, the ratios of 3’5’-substituted/3’-substituted and methoxylated/non-methoxylated anthocyanins were lower in ETH-treated (+L+E) grapes than in untreated grapes (+L-E), but in shaded grapes, the ratios of 3’5’-substituted/3’-substituted and methoxylated/non-methoxylated anthocyanins were significantly improved by ETH treatment (-L+E) related to no treatment (-L-E). The effect of ETH treatment on the ratio of acylated/non-acylated anthocyanins varied according to the sampling period and obviously, ETH treatment (+E) significantly decreased the ratio of acylated/non-acylated anthocyanins at 7 and 17 DAT. In summary, light exposure (+L) increased the ratio of 3’5’-substituted/3’-substituted anthocyanins and reduced the ratios of methoxylated/non-methoxylated and acylated/non-acylated anthocyanins. However, the effect of ETH treatment on the ratios of different modified anthocyanins depended on different light conditions. In general, ETH treatment of light-exposed grapes reduced the ratios of 3’5’-substituted/3’-substituted, methoxylated/non-methoxylated, and acylated/non-acylated anthocyanins, while the effects were opposite for shaded grapes.

We categorized non-anthocyanin compounds as flavanols, flavonols, or phenolic acids, and the ratios of the three non-anthocyanin phenolic substances are also shown in **Figure 6**. Light treatment (+L) significantly increased the ratio of flavonols but decreased the ratio of flavanols. ETH treatment (+E) did not affect the ratio of flavonols in either light-exposed (+L) or shaded (-L) grapes but did increase the ratio of flavanols in light-exposed (+L) grapes. The ratio of *p*-hydroxybenzoic acid was improved in light-exposed grapes shortly after ETH treatment (+L+E), but not in shaded (-L+E) grapes. In summary, light exposure increased the ratio of flavonols and decreased the ratio of flavanols. Exogenous ETH had no significant effect on flavonols but decreased the ratio of flavanols only in light-exposed (+L) grapes.

### 3.7 Differential effects of ethylene in light-exposed and shaded grapes

**Figure 7:**
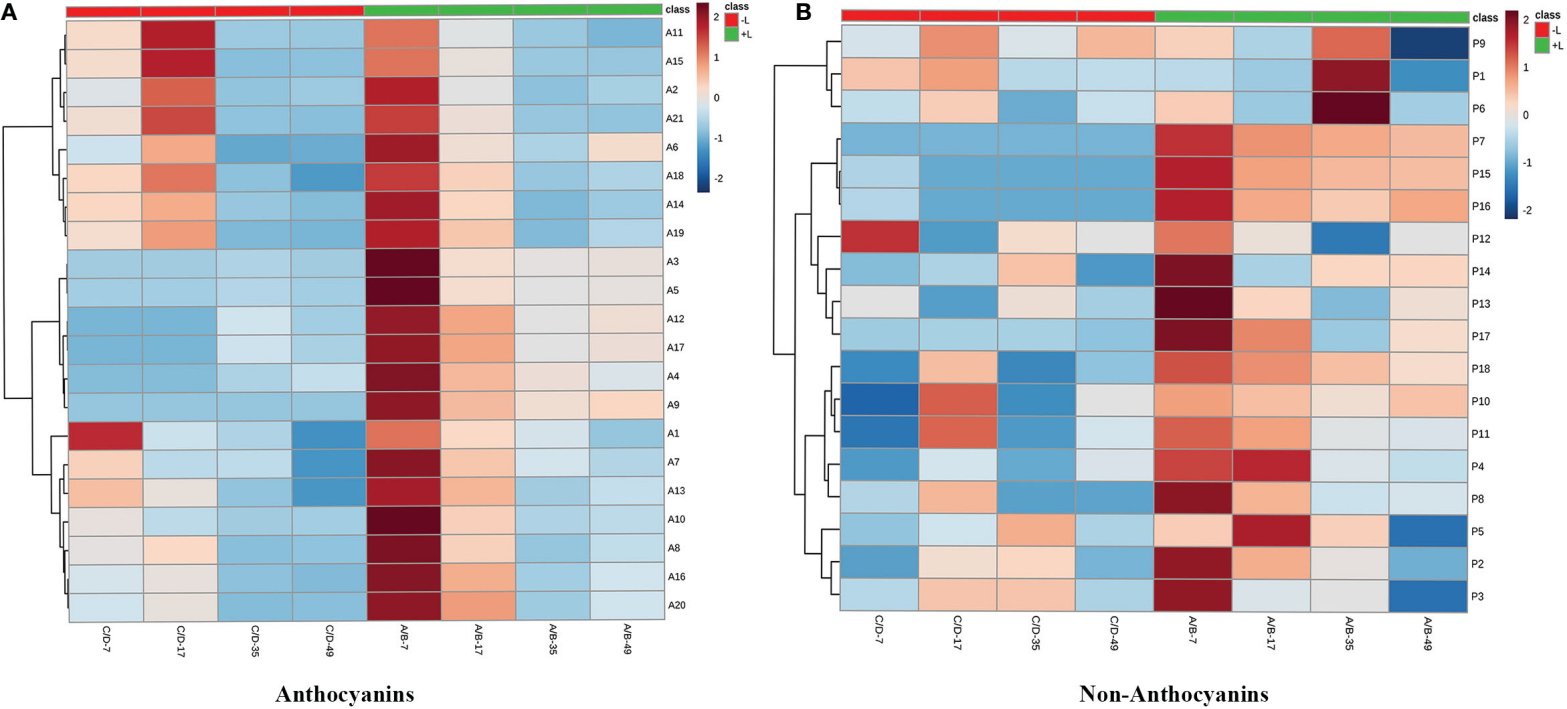
The heatmaps of increasing multiples of anthocyanin **(A)** and non-anthocyanin **(B)** compounds by ethylene treatments under light or dark.

To verify these observed differences in phenolic components, we used MetaboAnalyst to develop a heat map of the contents of anthocyanin and non-anthocyanin phenolic compounds (**Figure 7**). Each color in the figure represents an increasing multiple of the content of each anthocyanin compounds induced by ETH treatment under light or ETH treatment under dark conditions. As can be seen from the contrast between the red and blue shadows in the heatmap (**Figure 7A**), the increasing multiples for all individual anthocyanins (with the exception of malvidin-3-*O*-(cis-6-*O*-coumaryl)-glucoside (A1)) were significantly higher in light-exposed ETH-treated (+L+E) grapes at 7 DAT compared to ETH-treated shaded grapes (A/B-7 vs C/D-7). In addition, the increasing multiples of most anthocyanin compounds were also significantly higher in ETH-treated grapes under light (A/B) than under dark (C/D) at 17, 35, and 49 DAT, with the exception of malvidin-3-*O*-(6-*O*-acetyl)-glucoside (A11), malvidin-3-*O*-glucoside (A15), malvidin-3-*O*-(trans-6-*O*-coumaryl)-glucoside (A2), dephinidin-3-*O*-(trans-6-*O*-coumaryl)-glucoside (A6), dephinidin-3-*O*-glucoside (A18), peonidin-3-*O*-(6-*O*-acetyl)-glucoside (A14), and peonidin-3-*O*-glucoside (A19).

For non-anthocyanin compounds (**Figure 7B**), the increasing multiples for all non-anthocyanin compounds [with the exception of quercetin-3-*O*-glucuroside (P9), procyanin B1 (P1), procyanin C1 (P6), and syringetin-3-*O*-glucoside (P12)] were remarkably higher in ETH-treated light-exposed grapes (A/B) than in ETH-treated shaded grapes (C/D) at 7 and 17 DAT. At 35 and 49 DAT, the increasing multiples of most non-anthocyanin components (with the exception of syringetin-3-*O*-glucoside (P12), protocatechuic acid (P5), gallocatechin (P2), and epigallocatechin (P3)) were also significantly higher in ETH-treated light-exposed grapes (A/B) than in shaded grapes (C/D).

**Figure 8:**
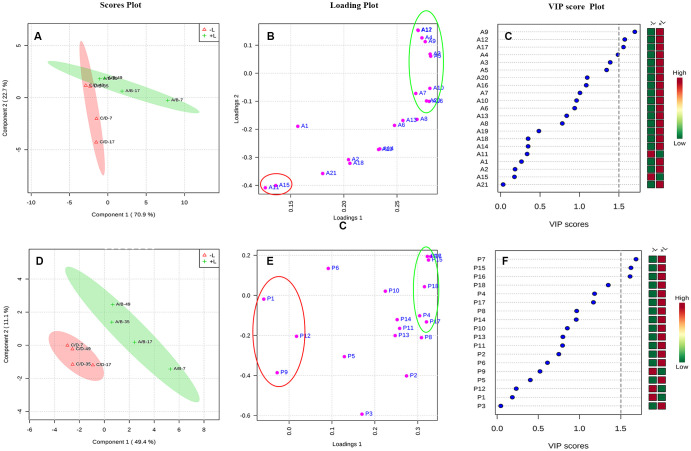
The PLS-DA analysis of increasing multiples of anthocyanin and non-anthocyanin compounds by ethylene treatments under light or dark. The +L and -L represent ethylene treatment under light and ethylene treatment under dark, respectively. Panel **(A–C)** are respectively, the scores plot, loading plot and VIP score plot for the anthocyanin compounds, and Panel **(D–F)** are respectively, the scores plot, loading plot and VIP score plot for the non-anthocyanin compounds.

Then, to further distinguish between the effects of ETH on light-exposed and shaded grapes and to detect potential biomarkers between the 2 groups, a partial least squares-discriminant analysis (PLS-DA) was conducted on the same data (**Figure 8**). As shown in **Figures 8A**, **D**, the ETH treatments (+L+E and –L+E) could be clearly separated. The major phenolic perturbations causing these discriminations were identified from the PLS-DA loading plots (**Figures 8B**, **E**) and the variable importance in the projection (VIP) plots demonstrated that certain identified variables contributed to the class separation. On these bases, the variables responsible for separating the ETH treatment under light (+L; A/B) and ETH treatment under dark (-L; C/D) were selected (**Figures 8C**, **F**). In our study, metabolites which had a VIP value greater than 1.5 were considered as the most relevant variables for explaining the difference, and these included 4 anthocyanin compounds (A9, A12, A17, A4) and 3 non-anthocyanin phenolic compounds (P7, P15, P16), therefore, cyanidin-3-*O*-(cis-6-*O*-coumaryl)-glucoside (A9), petunidin-3-*O*-(6-*O*-acetyl)-glucoside (A12), petunidin-3-*O*-glucoside (A17), petunidin-3-*O*-(trans-6-*O*-coumaryl)-glucoside (A4), myricetin-3-*O*-galactoside (P7), kaempferol-3-*O*-galactoside (P15), and kaempferol-3-*O*-glucoside (P16) were defined as main characteristic-differential components which had higher contents in light-exposed ETH-treated grapes (+L; A/B). However, malvidin-3-*O*-(6-*O*-acetyl)-glucoside (Mv-ac, A11), malvidin-3-*O*-glucoside (Mv, A15), quercetin-3-*O*-glucuroside (P9), procyanin B1 (P1), procyanin C1 (P6), and syringetin-3-*O*-glucoside (P12) also had a certain contribution rate in shaded ETH-treated grapes (-L; C/D). These results indicate that light influenced the effect of exogenous ETH on different phenolic individuals and cyanidin-3-*O*-(cis-6-*O*-coumaryl)-glucoside, petunidin-3-*O*-(6-*O*-acetyl)-glucoside, petunidin-3-*O*- (trans-6-*O*-coumaryl)-glucoside petunidin-3-*O*-glucoside, myricetin-3-*O*-galactoside, kaempferol-3-*O*-galactoside and kaempferol-3-*O*-glucoside were the major differential components between ethylene treatment under different light conditions.

## 4 Discussion

Here, we evaluated changes to the accumulation and composition of anthocyanins, phenolic acids, flavanols, and flavonols in Cabernet Sauvignon grapes. Our objective was to study the effects of light exposure and ethylene treatment on the ripening and phenolic composition of grapes in order to inform vineyard management for the improvement of wine. We identified three key takeaways: 1) that light influences the phenolic composition of wine grapes; 2) that ETH influences the phenolic composition of wine grapes; and 3) that light influences the effects of ETH on the phenolic composition of wine grapes.

Light has long been known to influence phenolic biosynthesis in grape berries (*V. vinifera* L.). Bunch shading caused a slight delay in berry ripening and reduced total soluble solids, but excessive sunlight exposure did not increase total soluble solids or anthocyanin accumulation (Chorti et al., 2010). Shading applied prior flowering resulted in greatly decreased flavonol concentration, but had little effect on berry development and ripening, including accumulation of anthocyanins and tannins (Downey et al., 2008; Chorti et al., 2010). In our study, the contents of all anthocyanin compounds were remarkably reduced when bunches were shaded (-L) at the onset of ripening. Similar reductions in anthocyanin accumulation in shaded berries have been previously reported (Zhang et al., 2012; Kondo et al., 2014). Furthermore, we observed that shading (-L) at the onset of ripening resulted in the inhibition of flavonol synthesis. Specifically, the contents of myricetin-3-*O*-glucoside, myricetin-3*-O*-glucoside, quercetin-3-*O*-glucoside, isorhamnetin-3-*O*-glucoside, and kaempferol-3-*O*-glucoside were significantly lower in shaded (-L) grapes than that in light-exposed (+L) grapes. Similar result have been reported that light exposure enhanced flavonol levels during grape berry development, as evidenced by the increased expression of the flavonol biosynthesis genes *VvFLS4*, *VvMYB12*, and *VvMYBF1* (Matus et al., 2009; Azuma and Yakushiji, 2012; Koyama et al., 2012). Shading increased the contents of flavanols like procyanin B1, procyanin C1, and catechin, while decreasing the contents of gallocatechin and epigallocatechin. These results are in agreement with previous research which found that shading decreased the relative abundance of trihydroxylated proanthocyanidin subunits (e.g., gallocatechin, epigallocatechin, and epicatechin-3-*O*-gallate) and the expression of 3’5’-hydroxylase (*VvF3’5’H*) (Koyama and Goto-Yamamoto, 2008; Koyama et al., 2012). However, the the effects of shading on flavanol levels were similar to the effects on flavonol levels, which is consistent with previous report (Downey et al., 2008).

In addition, bunch shading alters the ratios of phenolic compounds. We observed that light exposure (+L) increased the ratios of 3’5’-substituted/3’-substituted anthocyanins and flavonols. Other researchers also have found that shading can alter the anthocyanin compositions of fruits, including observing a greater proportion of the dioxygenated anthocyanins, as well as cyanidin and peonidin glucosides, in shaded berries (Koyama and Goto-Yamamoto, 2008), and increase the concentration of 3’-hydroxylated anthocyanins and decrease the concentration of 3’,5’-hydroxylated anthocyanins (Chorti et al., 2010), also the ratio of the transcript level of *VvF3’5’H* to that *VvF3’H* was observed decreased in shaded berries during pre-veraison periods (Koyama and Goto-Yamamoto, 2008), which made the 3’5’-substituted anthocyanins, including malvidin-3-*O*-glucoside, petunidin-3-*O*-glucoside, dephinidin-3-*O*-glucoside and their derivatives were more likely to be accumulated in berries exposed under light herein. In addition, we observed that the anthocyanin profiles of shaded (-L) grapes shifted toward acylated anthocyanins, in agreement with other studies (Haselgrove et al., 2000).

We found that the application of exogenous ETH altered the phenolic composition of grape berries. A growing body of evidence suggests that ETH signal transduction is required for non-climacteric fruit ripening. In the characteristically non-climacteric grape, ripening can be advanced by 2-chloroethylphosphonic acid (CEPA; an ETH-releasing reagent) and delayed by aminoethoxyvinylglycine (AVG; an inhibitor of ETH biosynthesis) (Böttcher et al., 2013). In addition, exogenous ETH has been found to stimulate sugar accumulation (Chervin et al., 2006), which was also observed in this study. ETH has also been shown to stimulate the transcription of structural genes (*VvUFGT*, *VvCHS*, *VvF3H*) and TFs (*VvMYBA1*) related to anthocyanin biosynthesis (El-Kereamy et al., 2003; Tira-Umphon et al., 2007). As a postharvest treatment, exogenous ETH has been found to increase the contents of phenols, anthocyanins, and aromatic compounds in wine obtained from treated grapes (Bellincontro et al., 2006; Becatti et al., 2010). In our study, the contents of all anthocyanin compounds were remarkably increased following treatment with exogenous ETH at the onset of ripening. These results are similar to previous reports of ETH-treated berries (Chervin et al., 2004; Liu et al., 2016).

In our previous study, we found that ethylene signals were involved in the regulation of phenolic biosynthesis, and involved in some steps of proanthocyanidin (PA) production, the PA content and transcriptions of *VvLAR1*, *VvLAR2*, *VvANR*, and *VvMYBPA1* were all increased after ethephon dipping, and exogenous ethylene also induced the non-anthocyanin accumulation in grape berry (Liu et al., 2016). Here, evaluated light- and ETH-induced changes in the concentrations of flavonols, flavanols, and phenolic acids and found that ETH treatment (+E) induced remarkable changes in flavonol compositions. Specifically, the concentrations of quercetin-3-*O*-glucoside, isorhamnetin-3-*O*-glucoside, and myricetin-3-*O*-glucoside were significantly higher in ETH-treated (+E) grapes. In addition, exogenous ETH decreased the ratio of flavanols and increased the ratio of *p*-hydroxybenzoic acid, likely due to the markedly increased flavonol content in treated grapes.

Perhaps most notably, we observed that light influences the effects of ETH on the phenolic composition of wine grapes. This is of particular importance as previous research has not reported this phenomenon. As mentioned, considerable amount of research effort has been focused on the effect of light on the triple response. In *Arabidopsis* seedlings, ETH suppresses hypocotyl elongation in darkness while promoting it in light. The HY5-COP1 light signaling module alters the transcription of downstream genes related to ETH signaling, thus participating in ETH- and light-regulated hypocotyl elongation (Zhong et al., 2009; Liang et al., 2012; Yu et al., 2013). Light controls the expression of key proteins in the ETH signaling pathway, including upregulating ETR1 and EIN4 and downregulating ETR2 and ERS2, also stimulates the accumulation of EIN3 in a COP1-dependent manner, while interfering with EIN3-mediated transcription (Zdarska et al., 2015). Numerous studies reports that light modulates ETH biosynthesis in many aspects of plant growth. For example, ETH production was 10-fold higher in light-grown *Arabidopsis* seedlings compared to etiolated *Arabidopsis* seedlings (Vogel et al., 1998). In ageratum, marigold, and salvia plug seedlings, ETH production depended upon different light qualities (Heo et al., 2009). In citrus fruit, phenylpropanoid and ETH biosynthesis were differentially regulated by blue LEDs (Ballester and Lafuente, 2017). These results suggest that the relationship between light and ETH signaling is complex. Using this concept, it was possible to discern the differential effects of ETH treatment on the phenolic composition of shaded and light-exposed grapes. In this study, we classified phenolic compounds as anthocyanins and non-anthocyanins according to their different sensitivities to exogenous ETH. At the onset of ripening, ETH treatment did not obviously affect the contents of anthocyanins and non-anthocyanins in shaded (-L) grapes, although a minor influence was observed in comparison to light-exposed (+L) grapes. In our previous study, we used real-time PCR (RT-PCR) to monitor the transcription of structural genes and TFs involved in phenolic biosynthesis, and our research revealed that ETH treatment promoted the accumulation of anthocyanins and proanthocyanidins in light-exposed (+L) grapes, and increased the expression of associated biosynthetic genes more effectively under light than under shading (Liu et al., 2016). Here, we also found that ETH treatment reduced the ratios of methoxylated/non-methoxylated, 3’5’-substituted/3’-substituted, and acylated/non-acylated anthocyanins and flavanols in light-exposed (+L) grapes, all of which were increased in shaded grapes (-L) treated with ETH (+E). We further used PLS-DA to discriminate between samples and found that individual anthocyanins (with the exception of malvidin-3-*O*-(6-*O*-acetyl)-glucoside and malvidin-3*-O*-glucoside) had a higher contribution rate for ETH treatment under light (+L). This is important because, due to their phenolic B ring substitution, methylated anthocyanins (e.g., peonidin, petunidin, and malvidin) and their derivatives are relatively stable and represent major anthocyanin pools (Roggero et al., 1986), so that in light-exposed condition, ETH treatment decreased the concentration of stable individual anthocyanins. While it is clear that light influences the effect of exogenous ETH on the phenolic composition of grapes, the molecular mechanism requires further clarification. The project contributes to the understanding of the impact of ethylene treatment under dark and light conditions on phenolic synthesis in grape berries. Furthermore, the outcomes are anticipated to enhance the scientific utilization of ethylene and facilitate the advancement of post-harvest storage technology.

## 5 Conclusions

Both light and exogenous ETH promoted ripening, improved the change in color from green to red and purplish red, and increased the contents of most anthocyanin and non-anthocyanin compounds in grape skin. Furthermore, light influenced the effect of ETH on the phenolic composition of grape skins. Specifically, ETH treatment had a more pronounced effect on the contents of phenolic compounds in light-exposed grapes compared to shaded grapes. In addition, ETH treatment decreased the ratios of methoxylated/non-methoxylated, 3’5’-substituted/3’-substituted, and acylated/non-acylated anthocyanins and flavanols in light-exposed grapes, all of which were increased by ETH treatment under dark conditions. Following ETH treatment, differences in phenolic compound composition between light-exposed and shaded grapes were reflected in 9 anthocyanin compounds and 6 non-anthocyanin phenolic compounds. The major differential components included cyanidin-3-*O*-(cis-6-*O*-coumaryl)-glucoside, petunidin-3-*O*-(6-*O*-acetyl)-glucoside, petunidin-3-*O*-(trans-6-*O*-coumaryl)-glucoside, petunidin-3-*O*-glucoside, myricetin-3-*O*-galactoside, kaempferol-3-*O*-galactoside, and kaempferol-3-*O*-glucoside. The future research aims to delve into the molecular mechanism that underlies the differential effect of ethylene treatment in light and dark conditions on phenolic synthesis in grape berries.

## Data availability statement

The original contributions presented in the study are included in the article/**Supplementary Material**. Further inquiries can be directed to the corresponding authors.

## Author contributions

ML: Data curation, Formal analysis, Writing – original draft, Investigation. QZ: Formal analysis, Methodology, Writing – review & editing. YY: Formal analysis, Investigation, Writing – original draft. QJ: Formal analysis, Investigation, Writing – original draft. HC: Supervision, Validation, Writing – review & editing, Formal analysis. ZZ: Funding acquisition, Resources, Writing – review & editing.
