# Supplementary material for: Light influences the effect of exogenous ethylene on the phenolic composition of Cabernet Sauvignon grapes
Source: Front Plant Sci. 2024 Feb 23;15:1356257. doi: 10.3389/fpls.2024.1356257 (PMC10920273; doi:10.3389/fpls.2024.1356257)
Supplement: Supplementary file 1 [file DataSheet_1.docx]

**Supplementary material captions:**

Supplemental Figure 1. Four designed experimental treatments

Supplemental Table 1. All used strandards and regression equation

Supplemental Table 2. The contents of anthocyanin monomers in berry skins between different treatments (μg/per berry). Different letters within a row for the same year indicate significant differences between treatments calculated by Duncan’s test (p < 0.05). [M^+^], molecular ion; [M−H]^+^, fragment ion.

Supplemental Table 3. The contents of non-anthocyanin monomers in berry skins between different treatments (μg/per berry). Different letters within a row for the same year indicate significant differences between treatments calculated by Duncan’s test (p < 0.05). [M^+^], molecular ion; [M−H]^+^, fragment ion.

Supplemental Table 4. Full name of the abbreviation for all individual anthocyanin

Supplemental Figure 1.


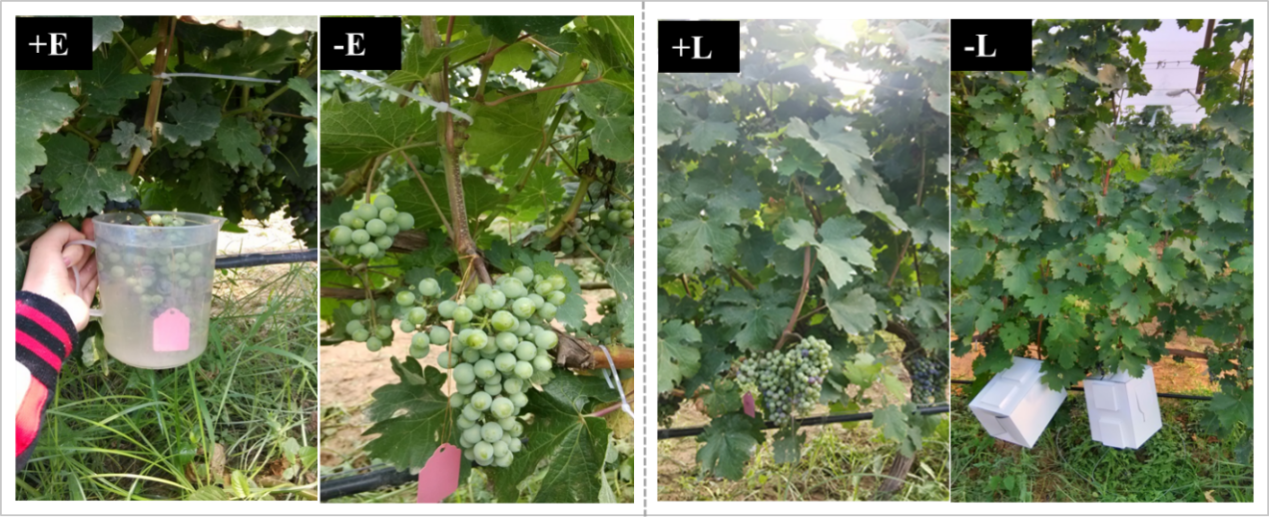


Supplemental Table 1.

| **Anthocyanins** | | **Regression equation** | **Non-Anthocyanins** | | **Regression equation** |
| --- | --- | --- | --- | --- | --- |
| A15 | Malvidin-3-*O*-glucoside | Y=0.0001X-0.4030 | P1 | Procyanin B1 | Y=0.0012X-0.4543 |
| A17 | Petunidin-3-*O*-glucoside | Y=0.0002X-0.5725 | P2 | Gallocatechin | Y=0.0045X+0.04 |
| A18 | Dephinidin-3-*O*-glucoside | Y=8E-05X-0.0008 | P3 | Epigallocatechin | Y=0.0006X+0.5294 |
| A19 | Peonidin-3-*O*-glucoside | Y=8E-05X+0.0239 | P4 | Catechin | Y=0.0054X-0.1845 |
| A20 | Cyanidin-3-*O*-glucoside | Y=7E-05X+0.1143 | P5 | Protocatechuic acid | Y=0.0018X-0.0035 |
|  |  |  | P6 | Procyanin C1 | Y=0.0176X-2.3421 |
|  |  |  | P7 | Myricetin-3-*O*-galactoside | Y=0.0004X-0.4083 |
|  |  |  | P8 | Myricetin-3-*O*--glucoside | Y=0.0002X-0.7003 |
|  |  |  | P9 | Quercetin-3-*O*--glucuronide | Y=0.0002X-1.0705 |
|  |  |  | P10 | Quercetin-3-*O*--galactoside | Y=0.0004X-0.0341 |
|  |  |  | P11 | Quercetin-3-*O*--glucoside | Y=0.0003X-1.2198 |
|  |  |  | P12 | Syringetin-3-*O*--glucoside | Y=0.0004X-0.0718 |
|  |  |  | P13 | Isorhamnetin-3-*O*--glucoside | Y=0.0006X-0.3867 |
|  |  |  | P14 | Quercetin-3-O--rhamnoside | Y=0.0007X-0.9103 |
|  |  |  | P15 | Kaempferol-3-*O*--galactoside | Y=0.0005X-0.4871 |
|  |  |  | P16 | Kaempferol-3-*O*--glucoside | Y=0.0003X-0.7302 |
|  |  |  | P17 | Myricetin | Y=0.0008X+0.1655 |

Supplemental Table 2.

| **Anthocyanins** | **[M^+^]/[M−H]^+^**  **(Frag.MS^2^*m*/*z*)** | **7 DAT** | | | | **17 DAT** | | | |  | **35 DAT** | | | **49 DAT** | | | |
| --- | --- | --- | --- | --- | --- | --- | --- | --- | --- | --- | --- | --- | --- | --- | --- | --- | --- |
|  |  | **+L+E** | **+L-E** | **-L+E** | **-L-E** | **+L+E** | **+L-E** | **-L+E** | **-L-E** | **+L+E** | **+L-E** | **-L+E** | **-L-E** | **+L+E** | **+L-E** | **-L+E** | **-L-E** |
| c-Mv-co (A1) | 639 (331) | 3.98±  0.25a | 1.49±  0.06b | 0.08±  0.02c | 0.02±  0.00d | 8.57±  0.87a | 4.84±  0.41b | 0.04±  0.01c | 0.03±  0.00c | 12.54±  0.50a | 9.55±  0.69b | 0.32±  0.04c | 0.34±  0.01c | 4.85±  0.77b | 6.62±  0.76a | 0.00±  0.00d | 0.24±  0.01c |
| t-Mv-co (A2) | 639 (331) | 40.01±  0.47a | 11.94±  1.66b | 1.58±  0.05c | 0.91±  0.04d | 83.35±  5.73a | 46.58±  2.99b | 5.32±  0.26c | 1.83±  0.09d | 117.14±10.75a | 98.09±  10.16a | 13.72±  1.75b | 11.27±  1.92b | 92.90±  4.65a | 67.75±5.21b | 17.78±  0.30c | 13.84±  0.23d |
| c-Pt-co (A3) | 625 (317) | 0.62±  0.07a | 0.11±  0.01b | 0.00±  0.00c | 0.00±  0.00c | 0.76±  0.07a | 0.45±  0.06b | 0.00±  0.00c | 0.00±  0.00c | 0.85±  0.06a | 0.63±  0.04b | 0.01±  0.00c | 0.01±  0.00c | 0.29±  0.06a | 0.20±  0.02b | 0.00±  0.00c | 0.00±  0.00c |
| t-Pt-co  (A4) | 625 (317) | 7.50±  0.25a | 1.81±  0.05b | 0.00±  0.00c | 0.00±  0.00c | 11.29±  0.44a | 5.42±  0.25b | 0.03±  0.01c | 0.00±  0.00d | 11.12±  1.43c | 7.96±  0.37b | 0.27±  0.02d | 0.46±  0.17c | 4.77±  0.73a | 4.22±  1.23a | 0.44±0.02b | 0.51±  0.11b |
| c-Dp-co (A5) | 611 (303) | 0.16±  0.01a | 0.02±  0.00b | 0.00±  0.00c | 0.00±  0.00c | 0.22±  0.01a | 0.11±  0.02b | 0.00±  0.00c | 0.00±  0.00c | 0.18±  0.04a | 0.12±  0.01b | 0.01±  0.00c | 0.01±  0.00c | 0.09±  0.02a | 0.06±  0.00b | 0.00±  0.00c | 0.00±  0.00c |
| t-Dp-co (A6) | 611 (303) | 2.24±  0.08a | 0.50±  0.05b | 0.04±  0.00c | 0.02±  0.00d | 3.17±  0.09a | 1.48±  0.09b | 0.08±  0.01c | 0.03±  0.00d | 2.58±  0.49a | 1.92±  0.12b | 0.09±  0.02c | 0.13±  0.02c | 1.85±  0.60a | 0.82±  0.02b | 0.10±  0.01c | 0.14±  0.03c |
| c-Pn-co (A7) | 609 (301) | 1.23±  0.13a | 0.33±  0.04b | 0.01±  0.00c | 0.01±  0.00c | 1.47±  0.16a | 0.74±  0.02b | 0.01±  0.00c | 0.01±  0.00c | 1.52±  0.11a | 1.17±  0.05b | 0.07±  0.01c | 0.06±  0.01c | 0.86±  0.18a | 0.88±  0.11a | 0.00±  0.00c | 0.03±  0.00b |
| t-Pn-co (A8) | 609 (301) | 10.78±  0.17a | 2.48±  0.13b | 0.43±  0.04c | 0.22±  0.03d | 15.18±  0.97a | 6.40±  0.28b | 0.92±  0.07c | 0.40±  0.02d | 14.87±  1.61a | 12.37±  1.31a | 1.74±  0.16b | 1.54±  0.45b | 11.31±  2.71a | 6.99±  0.86b | 2.66±  0.12c | 2.27±  0.18d |
| c-Cy-co (A9) | 595 (287) | 0.14±  0.01a | 0.04±  0.00b | 0.00±  0.00c | 0.00±  0.00c | 0.12±  0.03a | 0.07±  0.01b | 0.00±  0.00c | 0.00±  0.00c | 0.10±  0.01a | 0.09±  0.01a | 0.00±  0.00b | 0.00±  0.00b | 0.05±  0.00a | 0.04±  0.00b | 0.00±  0.00c | 0.00±  0.00c |
| t-Cy-co (A10) | 595 (287) | 1.43±  0.05a | 0.35±  0.03b | 0.09±  0.01c | 0.05±  0.00d | 1.61±  0.11a | 0.78±  0.07b | 0.12±  0.01c | 0.10±  0.02c | 1.21±  0.18a | 1.05±  0.05a | 0.10±  0.01b | 0.09±  0.00b | 0.88±  0.15a | 0.70±  0.17a | 0.12±  0.01b | 0.11±  0.00b |
| Mv-ac (A11) | 535 (331) | 150.16±3.69a | 56.17±1.96b | 5.65±  0.06c | 2.95±  0.03d | 269.46±16.75a | 162.59±  17.79b | 16.35±  0.32c | 5.10±  0.65d | 411.65±  65.77a | 353.92±  24.56a | 56.73±  7.44b | 47.9±  4.68b | 267.80±  17.47a | 270.86±14.50a | 67.72±  1.42b | 58.32±  4.79c |
| Pt-ac  (A12) | 521 (317) | 30.38±  1.22a | 8.14±  0.32b | 0.00±  0.00c | 0.00±  0.00c | 36.22±  0.79a | 16.26±  1.80b | 0.04±  0.00c | 0.00±  0.00d | 29.35±  5.71a | 24.11±  1.03a | 0.39±  0.05b | 0.39±  0.04b | 23.92±  2.56a | 16.48±  2.92b | 1.07±  0.06d | 1.85±  0.06c |
| Dp-ac (A13) | 493 (331) | 8.89±  0.21a | 2.41±  0.01b | 0.04±  0.01c | 0.02±  0.00d | 10.41±  0.21a | 4.29±  0.57b | 0.07±  0.01c | 0.04±  0.00d | 7.07±  1.73a | 5.98±  0.26a | 0.14±  0.03b | 0.13±  0.01b | 6.51±  1.36a | 4.44±  0.99a | 0.34±  0.01d | 0.59±  0.01c |
| Pn-ac  (A14) | 505 (301) | 23.17±  0.56a | 6.63±  0.23b | 1.05±  0.02c | 0.51±  0.00d | 26.76±  0.96a | 12.91±  1.73b | 1.95±  0.09c | 0.80±  0.11d | 26.21±  3.35a | 25.79±  2.70a | 4.80±  0.43b | 4.10±  0.76b | 23.66±  6.56a | 19.35±  4.08a | 6.25±  0.17b | 5.91±  0.95b |
| Mv  (A15) | 493 (331) | 156.79±3.53a | 51.04±1.79b | 2.96±  0.04c | 1.42±  0.00d | 238.64±8.99a | 125.47±  15.21b | 7.35±  0.24c | 1.97±  0.22d | 310.31±  35.15a | 267.32±  12.92a | 27.03±  3.86b | 26.0±  2.00b | 237.35±  10.82a | 210.21±28.86a | 39.55±  0.71b | 37.43±  2.15b |
| Cy-ac (A16) | 491 (287) | 2.98±  0.03a | 0.81±  0.04b | 0.06±  0.01c | 0.05±  0.00c | 2.28±  0.04a | 1.00±  0.12b | 0.09±  0.01c | 0.06±  0.01d | 1.30±  0.28a | 1.35±  0.06a | 0.10±  0.02b | 0.13±  0.02b | 1.61±  0.27a | 1.19±  0.17b | 0.15±  0.01d | 0.21±  0.01c |
| Pt  (A17) | 479 (317) | 42.67±  0.77a | 11.11±0.42b | 0.00±  0.00c | 0.00±  0.00c | 51.28±  1.60a | 22.49±  2.66b | 0.04±  0.01c | 0.00±  0.00d | 47.91±  7.96a | 38.26±  1.62b | 0.62±  0.05c | 0.62±  0.04c | 39.95±  1.82a | 28.01±  4.73a | 1.68±  0.09c | 2.61±  0.05b |
| Dp  (A18) | 465 (303) | 24.22±  0.27a | 6.37±  0.25b | 0.08±  0.00c | 0.03±  0.00d | 28.64±  1.33a | 11.95±  1.69b | 0.18±  0.03c | 0.06±  0.01d | 22.30±  4.63a | 18.99±  0.82a | 0.35±  0.04b | 0.33±  0.03b | 20.43±  5.09a | 14.66±  2.76a | 0.88±  0.02c | 1.39±  0.02b |
| Pn  (A19) | 463 (301) | 37.28±  0.75a | 10.91±0.36b | 0.97±  0.03c | 0.49±  0.02d | 36.08±  1.28a | 14.69±  2.40b | 1.46±  0.08c | 0.57±  0.09d | 34.14±  4.59a | 31.40±  2.14a | 4.09±  0.37b | 3.79±  0.68b | 35.60±  6.06a | 25.08±  1.12b | 6.43±  0.19c | 6.18±  0.19c |
| Cy  (A20) | 449 (287) | 6.96±  0.13a | 1.92±  0.07b | 0.13±  0.02c | 0.10±  0.01c | 5.41±  0.03a | 2.02±  0.37b | 0.16±  0.01c | 0.10±  0.01d | 3.46±  0.75a | 3.61±  0.16a | 0.23±  0.03b | 0.30±0.04b | 4.78±  0.75a | 3.57±  0.87a | 0.37±  0.01c | 0.46±  0.03b |
| TC  (A21) |  | 551.57±8.69a | 174.59±6.37b | 13.17±0.21c | 6.81±  0.16d | 830.92±33.08a | 442.12±  44.61b | 34.22±  0.54c | 11.09±  0.98d | 1055.80±121.16a | 903.69±  53.15a | 110.82±  13.94b | 97.6±  5.17b | 779.46±  32.55a | 682.18±  52.02b | 145.55±  6.69c | 132.10±8.13c |

Supplemental Table 3.

| **Non-Anthocyanins** | **[M^+^]/[M−H]^+^**  **(Frag.MS^2^*m*/*z*)** | **7 DAT** | | | | **17 DAT** | | | |  | **35 DAT** | | | **49 DAT** | | | |
| --- | --- | --- | --- | --- | --- | --- | --- | --- | --- | --- | --- | --- | --- | --- | --- | --- | --- |
|  |  | **+L+E** | **+L-E** | **-L+E** | **-L-E** | **+L+E** | **+L-E** | **-L+E** | **-L-E** | **+L+E** | **+L-E** | **-L+E** | **-L-E** | **+L+E** | **+L-E** | **-L+E** | **-L-E** |
| Procyanin B1  (P1) | 639 (331) | 3.78±  0.32b | 3.80±  0.30b | 4.86±  0.42a | 4.19±  0.41a | 1.80±  0.11b | 1.89±  0.09b | 2.73±  0.21a | 2.23±  0.18a | 2.86±  0.21a | 1.99±  0.17b | 2.71±  0.22a | 2.74±  0.19a | 3.30±  0.29a | 3.95±  0.41a | 3.81±  0.32a | 3.79±  0.30a |
| Gallocatechin  (P2) | 639 (331) | 0.10±  0.00d | 0.04±  0.01c | 0.11±  0.01b | 0.14±  0.01a | 0.19±  0.02a | 0.12±  0.01b | 0.09±  0.01c | 0.07±  0.01c | 0.27±  0.02a | 0.21±  0.02b | 0.14±  0.01c | 0.10±  0.01d | 0.54±  0.04b | 0.64±  0.05a | 0.37±  0.03d | 0.43±  0.04c |
| Epigallocatechin  (P3) | 625 (317) | 0.02±  0.00a | 0.02±  0..00a | 0.02±  0.00a | 0.02±  0.00a | 0.03±  0.00a | 0.02±  0.00b | 0.02±  0.00b | 0.01±  0.00c | 0.03±  0.00a | 0.03±  0.00a | 0.02±  0.00b | 0.02±  0.00b | 0.04±  0.00a | 0.04±  0.00a | 0.02±  0.00b | 0.02±  0.00b |
| Catechin  (P4) | 625 (317) | 0.67±  0.04b | 0.54±  0.04c | 0.85±  0.06a | 0.99±  0.08a | 0.25±  0.02b | 0.20±  0.02c | 0.63±  0.05a | 0.63±  0.04a | 0.22±  0.02a | 0.21±  0.02a | 0.20±  0.02a | 0.23±  0.02a | 0.34±  0.03b | 0.35±  0.03b | 0.43±  0.03a | 0.43±  0.03a |
| Protocatechuic acid  (P5) | 611 (303) | 0.01±  0.00a | 0.00±  0.00a | 0.00±  0.00a | 0.00±  0.00a | 0.01±  0.00a | 0.00±  0.00b | 0.00±  0.00b | 0.00±  0.00b | 0.01±  0.00a | 0.00±  0.00b | 0.00±  0.00b | 0.00±  0.00b | 0.00±  0.00b | 0.01±  0.00a | 0.00±  0.00b | 0.00±  0.00b |
| Procyanin C1  (P6) | 611 (303) | 1.37±  0.11a | 1.16±  0.08b | 1.32±  0.09ab | 1.42±  0.11a | 0.36±  0.03a | 0.44±  0.04b | 1.20±  0.10a | 1.02±  0.10a | 0.64±  0.05a | 0.35±  0.03c | 0.49±  0.04b | 0.69±  0.05a | 0.42±  0.04b | 0.50±  0.04b | 0.65±  0.05a | 0.69±  0.06a |
| Myricetin-3-*O*-galactoside (P7) | 609 (301) | 0.32±  0.02a | 0.17±  0.01b | 0.00±  0.00c | 0.00±  0.00c | 0.68±  0.05a | 0.47±  0.03b | 0.00±  0.00c | 0.00±  0.00c | 1.60±  0.12a | 1.19±  0.10b | 0.00±  0.00c | 0.00±  0.00c | 1.48±  0.11a | 1.20±  0.09b | 0.00±  0.00c | 0.00±  0.00c |
| Myricetin-3-*O*--glucoside  (P8) | 609 (301) | 1.60±  0.09a | 0.77±  0.06b | 0.04±  0.00c | 0.04±  0.00c | 3.18±  0.03a | 2.08±  0.02b | 0.05±  0.01c | 0.03±  0.00d | 5.41±  0.42a | 4.55±  0.39b | 0.12±  0.01c | 0.13±  0.01c | 5.33±  0.49a | 4.36±  0.37b | 0.14±  0.11c | 0.16±  0.09c |
| Quercetin-3-*O*--glucuroside (P9) | 595 (287) | 6.59±  0.51a | 6.08±  0.42a | 6.55±  0.50a | 6.39±  0.39a | 5.82±  0.49a | 5.91±  0.39a | 4.52±  0.41b | 3.94±  0.32b | 5.77±  0.41a | 4.88±  0.32b | 4.34±  0.41c | 4.22±  0.41c | 4.77±  0.40a | 5.95±  0.51a | 3.79±  0.31b | 3.41±  0.29b |
| Quercetin-3-*O*--galactoside (P10) | 595 (287) | 1.53±  0.12a | 1.07±  0.08b | 0.83±  0.07c | 0.90±  0.07c | 1.98±  0.11a | 1.45±  0.10b | 0.56±  0.05c | 0.37±  0.03d | 4.25±  0.03b | 3.28±  0.03c | 0.52±  0.04a | 0.52±  0.04a | 4.55±  0.39a | 3.34±  0.27b | 0.64±  0.05c | 0.51±  0.04d |
| Quercetin-3-*O*--glucoside (P11) | 535 (331) | 4.51±  0.37a | 2.95±  0.21b | 1.96±  0.16c | 2.06±  0.16c | 6.20±  0.52a | 4.33±  0.41b | 1.33±  0.12c | 0.88±  0.07d | 11.45±  0.10a | 9.14±  0.08b | 1.26±  0.10c | 1.22±  0.12c | 11.52±  1.07a | 9.30±  0.92b | 1.60±  0.14c | 1.30±  0.18c |
| Syringetin-3-*O*--glucoside (P12) | 521 (317) | 0.27±  0.02a | 0.16±  0.01b | 0.04±  0.01c | 0.02±  0.00d | 1.02±  0.01a | 0.74±  0.06b | 0.07±  0.01c | 0.07±  0.01c | 2.70±  0.22a | 2.72±  0.20a | 0.49±  0.43b | 0.35±  0.30c | 3.75±  0.31a | 2.78±  0.23b | 0.61±  0.05c | 0.45±  0.04d |
| Isorhamnetin-3-*O*--glucoside (P13) | 493 (331) | 0.83±  0.06a | 0.25±  0.02b | 0.03±  0.00c | 0.02±  0.00c | 1.54±  0.12a | 0.83±  0.07b | 0.03±  0.00c | 0.04±0.01c | 3.66±  0.32a | 3.73±  0.30a | 0.10±  0.01b | 0.06±  0.01c | 5.21±  0.44a | 3.03±  0.31b | 0.14±  0.01c | 0.12±  0.01c |
| Quercetin-3-*O*--rhamnoside (P14) | 505 (301) | 0.41±  0.03a | 0.17±  0.01b | 0.04±  0.00d | 0.09±  0.01c | 0.16±  0.02b | 0.25±  0.02a | 0.00±  0.00c | 0.00±  0.00c | 0.27±  0.02a | 0.23±  0.02a | 0.09±  0.01b | 0.07±  0.01b | 0.24±  0.02a | 0.20±  0.02a | 0.00±  0.00b | 0.00±  0.00b |
| Kaempferol-3-*O*--galactoside (P15) | 493 (331) | 0.19±  0.02a | 0.09±  0.01b | 0.03±  0.00d | 0.05±  0.01c | 0.28±  0.02a | 0.18±  0.02b | 0.01±  0.00c | 0.00±  0.00c | 0.68±  0.05a | 0.48±  0.04b | 0.01±  0.00c | 0.00±  0.00c | 0.60±  0.01a | 0.44±  0.01b | 0.00±  0.00c | 0.00±  0.00c |
| Kaempferol-3-*O*--glucoside (P16) | 491 (287) | 0.39±  0.03a | 0.17±  0.02b | 0.03±  0.00d | 0.05±  0.01c | 0.53±  0.04a | 0.34±  0.03b | 0.00±  0.00c | 0.00±  0.00c | 1.33±  0.11a | 1.01±  0.09b | 0.01±  0.00c | 0.00±  0.00c | 1.38±  0.12a | 0.89±  0.06b | 0.00±  0.00c | 0.00±  0.00c |
| Myricetin  (P17) | 479 (317) | 0.04±  0.00a | 0.02±  0.00b | 0.01±  0.00c | 0.01±  0.00c | 0.06±  0.01a | 0.03±  0.00b | 0.01±  0.00c | 0.01±  0.00c | 0.07±  0.01a | 0.06±  0.01a | 0.01±  0.00b | 0.01±  0.00b | 0.09±  0.01a | 0.06±  0.01b | 0.02±  0.00c | 0.01±  0.00d |
| Totalcontent  (P18) | 465 (303) | 22.63±  0.21a | 17.46±0.14b | 16.72±0.11c | 16.39±0.09c | 24.09±  2.11a | 19.28±  1.72b | 11.26±1.22c | 9.30±  0.99c | 41.20±  3.88a | 34.08±  2.98b | 10.51±  0.95c | 10.35±  0.89c | 43.55±  3.02a | 37.01±  2.14b | 12.22±  1.19c | 11.33±  1.02c |

Supplemental Table 4.

| Tag | Abbreviation | Full name |
| --- | --- | --- |
| A1 | c-Mv-co | Malvidin-3-*O*-(*cis*-6-*O*-coumaryl)-glucoside |
| A2 | t-Mv-co | Malvidin-3-*O*-( *trans*-6-*O*-coumaryl)-glucoside |
| A3 | c-Pt-co | Petunidin-3-*O*-(*cis*-6-*O*-coumaryl)-glucoside |
| A4 | t-Pt-co | Petunidin-3-*O*-(*trans*-6-*O*-coumaryl)-glucoside |
| A5 | c-Dp-co | Dephinidin-3-*O*-(*cis*-6-*O*-coumaryl)-glucoside |
| A6 | t-Dp-co | Dephinidin-3-*O*-( *trans*-6-*O*-coumaryl)-glucoside |
| A7 | c-Pn-co | Peonidin-3-*O*-(*cis*-6-*O*-coumaryl)-glucoside |
| A8 | t-Pn-co | Peonidin-3-*O*-(*trans*-6-*O*-coumaryl)-glucoside |
| A9 | c-Cy-co | Cyanidin-3-*O*-(*cis*-6-*O*-coumaryl)-glucoside |
| A10 | t-Cy-co | Cyanidin-3-*O*-( *trans*-6-*O*-coumaryl)-glucoside |
| A11 | Mv-ac | Malvidin-3-*O*-(6-*O*-acetyl)-glucoside |
| A12 | Pt-ac | Petunidin-3-*O*-(6-*O*-acetyl)-glucoside |
| A13 | Dp-ac | Dephinidin-3-*O*-(6-*O*-acetyl)-glucoside |
| A14 | Pn-ac | Peonidin-3-*O*-(6-*O*-acetyl)-glucoside |
| A15 | Mv | Malvidin-3-*O*-glucoside |
| A16 | Cy-ac | Cyanidin-3-*O*-(6-*O*-acetyl)-glucoside |
| A17 | Pt | Petunidin-3-*O*-glucoside |
| A18 | Dp | Dephinidin-3-*O*-glucoside |
| A19 | Pn | Peonidin-3-*O*-glucoside |
| A20 | Cy | Cyanidin-3-*O*-glucoside |
